# Mechanistic toxicity profiling of nicotine-rich e-liquids: mitochondrial and oxidative stress responses in ALI-exposed bronchial cells

**DOI:** 10.1007/s11010-025-05430-9

**Published:** 2025-11-19

**Authors:** Rosalia Emma, Alfio Distefano, Pietro Zuccarello, Chiara Copat, Margherita Ferrante, Gea Marzia Oliveri Conti, Roberta Pulvirenti, Giuseppe Carota, Davide Campagna, Riccardo Polosa, Sonja Rust, Massimo Caruso, Giovanni Li Volti

**Affiliations:** 1 Department of Medicine and Surgery, “Kore” University of Enna, Contrada Santa Panasia, 94100 Enna, Italy; 2 Department of Biomedical and Biotechnological Sciences, University of Catania, Via S. Sofia, 97, 95123 Catania, Italy; 3 Department of Psychology and Health Sciences, Pegaso Telematic University, 80143 Naples, Italy; 4 Department of Medical, Surgical Sciences and Advanced Technologies “G.F. Ingrassia”, University of Catania, Via S. Sofia, 87, 95123 Catania, Italy; 5 Center of Excellence for the Acceleration of Harm Reduction, University of Catania, Via S. Sofia, 97, 95123 Catania, Italy; 6 Department of Clinical and Experimental Medicine, University of Catania, Via S. Sofia, 97, 95123 Catania, Italy; 7 ECLAT Srl, University of Catania, Via S. Sofia, 89, 95123 Catania, Italy

**Keywords:** Tobacco smoke, Electronic cigarette, Cytotoxicity, Microplastics, Metals, Mitochondrial potential

## Abstract

Combustion byproducts contained in cigarette smoke are considered the main responsible for the occurring of serious chronic diseases. Aerosols from electronic cigarettes contain substantially fewer of these dangerous byproducts, potentially reducing health risks. This study examined the potential of four different commercial nicotine-rich e-liquids to induce cytotoxicity and mitochondrial dysfunction and oxidative stress in human bronchial epithelial cells (H292), using air-liquid interface (ALI) exposure. In addition to a qualitative control of the e-liquids, by dosing contaminants, we assessed cell viability, apoptosis, mitochondrial membrane potential, and reactive oxygen species (ROS) generation, by comparing e-cig aerosols to 1R6F reference cigarette smoke. All tested e-liquids showed very low levels of trace metals and microplastics, with contaminant concentrations below WHO drinking water limits. Furthermore, e-liquid aerosol induced significantly reduced cytotoxicity compared to 1R6F regular cigarette smoke, and mitochondrial integrity was preserved. Furthermore, no ROS generation was observed when using flavored e-cigarette aerosol. These results provide evidence of the lower potential toxicity of e-cigarettes compared to tobacco cigarettes in an in vitro model simulating real-world smoke exposure.

## Introduction

Electronic cigarettes (e-cigarettes) are a worldwide consumer product essentially consisting of a battery powered electronic devices that operate by heating an element (most commonly, a metal coil), that vaporizes a solution (e-liquid), mainly consisting of glycerol, propylene glycol (PG), distilled water, and flavorings, and which may or may not contain nicotine [1].

Considering the composition of the liquids, the lack of combustion and the results of chemical analyzes reported in previous studies [2–4], it could be hypothesized that the aerosol deriving from the heating of these e-liquids is free of tar and carbon monoxide, and in general less harmful to human health. Based on this assumption, e-cigarettes could be considered a useful tool for smoking harm reduction. Science is expected to prove these hypotheses, but the lack of standardization in studies, especially those conducted in vitro, often provide conflicting results, generating confusion for consumers and regulatory bodies. Analysis-wise, these products are hardly comparable to tobacco cigarettes. Therefore, it is necessary to establish standardized protocols for a simple and fast analysis of the composition of e-liquids, the aerosol and the toxicological effects of the aerosol on human cells in vitro. The variety of formulation of the e-liquids used to generate the aerosol on the market is an issue to be taken into due account [1].

In this regard, our group has been previously engaged in the definition of a test panel based on standardized regimens that can provide a basic toxicological evaluation of the products available on the market [5]. In the present work, we analyzed the safeness of different e-liquids typically widespread in the Italian market, highlighting their impact both on the cellular redox status and the mitochondrial functionality. The industrial manufacturing process of e-liquids involves their contact with metal and plastic parts; after production, they are stored and shipped in plastic bottles, normally darkened to protect the photosensitive nicotine. Production and storage processes could lead to the presence of metal residues, such as arsenic, lead, aluminum, iron, mercury and cadmium, or nanoplastics (NPs) and microplastics (MPs) in the liquids themselves, which could compromise their quality and safety for consumers [5]. In particular, transition metals (e.g., Zn, Ni, Cu) can catalyze redox reactions and generate reactive oxygen species (ROS), while inhaled microplastics may cause mechanical irritation or act as carriers for toxicants [6–8]. Indeed, these components could act as relevant contributors to the observed oxidative and mitochondrial effects.

Therefore, in addition to the evaluation of metal and MPs content, we tested the ability of the aerosol to produce ROS upon heating, in order to assess the purity of the e-liquids with respect to these undesirable contaminants. We also evaluated the ability of the aerosol to induce cytotoxicity on human pulmonary epithelial cells, leading to mitochondrial malfunctions.

Despite cytotoxicity tests are considered essential to establish the safety for this kind of products, giving particular focus on toxicity induced by cigarette smoke on human cells, there is lack of specific indications for performing these tests after exposure to e-cigarette aerosol. For this reason, the use of multiple and alternative methods of safety evaluation, in addition to Neutral Red Uptake (NRU) assay, which still remains the benchmark test, was considered in order to propose consistent and reliable results [9]. 

Moreover, following strict and rigorous methodological conditions, which include exposure method and regimens, represents one of the most critical parts of the analysis in this field. Due to the physiological behavior of lung epithelial cells in presence of smoke and aerosols, the employ of an air-liquid interface model of exposure allowed to maintain a translational model and, consequently, to ensure an appropriate evaluation of the cell conditions. The last point to be considered was the regimen of exposure of the cells to the smoke/aerosol: given the need for reproducibility of the study, the cells were exposed to cigarette smoke and e-cigarette aerosol with standardized and reproducible regimens, such as Health Canada Intense and CORESTA Reference Method 81 regimens.

## Materials and methods

### Nano and microplastic analysis

Nano- and micro-plastics analyses were carried out according to a method patented in our laboratories and previously described in Ferrante et al. [10]. Briefly, after homogenization of samples by vortex, an aliquot of 10 ml for each e-liquid was added to nitric acid 65% and mineralization of the samples was performed in an open vessel at 60 °C for 24 h. Subsequently samples were washed by 10 ml of ultrapure water and 10 ml of dichloromethane and centrifuged at 4000 rpm for 5 min. The solvent was dispersed on an aluminum and copper alloy stub with a diameter of 25 mm through nebulization by a nebulizer. Then, stubs were coated with gold and samples were ready to SEM-EDX analysis. The counting method was applied to an overall reading area within the stub for a total of 228 fields at magnification of 1500x, corresponding to 1.0 mm^2^. Micro-analytical acquisition for recognition of the particles containing only carbon and determination of particle size and counting were performed.

### Trace elements analysis

Aliquots of 1 mL of each e-liquid was digested with 3 mL of Nitric Acid 65%, Suprapur^®^ for trace analysis (Carlo Erba Reagents, Milan, Italy) in a Microwave Ethos TC (Milestone, Sorisole, BG, Italy), equipped with pressurized Teflon vessels. The digestion was performed stepwise up to 200 °C in 10 min (1000 W), followed by a 15 min rest at 200 °C (1000 W). At the end of mineralization, the digested samples were transferred into graduated polypropylene tubes and diluted to 20 mL using Milli-Q water and filtrated by a 0.45 μm nylon filters before analysis. Trace elements were quantified with an Inductively Coupled Plasma – Mass Spectrometer (ICP-MS Elan DRCe, Perkin Elmer USA). The instrument calibration was performed by using the standard addition technique to minimize matrix effects, covering a concentration range from 0.5 to 10 µg/L, and a 25 µg/L concentration of Y as internal standard. Mono-elemental certified standards (1000 mg/L) were purchased from CPAchem S.r.l. (Rome, Italy). The limit of detection (LOD µg/L) were calculated by analyzing ten acid extract blanks based on the mean ± 3 SD/mean ± 10 SD criterion. They resulted as follows: Al < 4.4; As < 1.1; B < 18; Cd < 0.1; Cr < 0.5; Cu < 1.1; Fe < 6; Hg < 0.5; Mn < 1.5; Ni < 1.0; Pb < 0.5; Sb < 0.5; Se < 0.5; V < 1.1. As quality controls, each sample was spiked at 10 µg/L before digestion. The recoveries calculated are in the range 91–118%.

### Cell culture

Human bronchial epithelial cells (NCI-H292, ATCC^®^ CRL-1848™) were selected due to their wide use as a standardized in vitro human bronchial epithelial model, offering reproducibility, robust adherence, and compatibility with the experimental setup. Cells were cultured as previously described [11]. Briefly, H292 cells, were cultured in RPMI 1640 medium (10% fetal bovine serum, 1% penicillin/streptomycin, 2 mM L-glutamine) at 37 °C, 5% CO_2_ in a humidified atmosphere. Then, cells were seeded in 12 mm Transwell^®^ inserts (Corning Incorporated, NY, USA) at a density of 3 × 10^5^ cells/ml sustained by 0.7 ml of RPMI 1640 medium in the basal compartment of each well and 0.5 ml in the apical compartment of each Transwell^®^ insert, 48 h prior to exposure. Cell starvation was done 24 h prior to exposure by replacing the basal and apical medium with 0.7 and 0.5 mL respectively of RPMI 1640 medium containing 2 mM glutamine, 1% penicillin/streptomycin. Next, when the 80% confluency was reached, the apical medium was removed from each insert and two inserts per test product were transitioned to the exposure chamber with 25 ml of DMEM-high glucose (DMEM-hg) in the basal compartment in order to perform the air-liquid interface (ALI) exposure. For each smoking/vaping regime, one exposure chamber was connected to the LM4E port without the device in order to expose H292 cells to laboratory air filtered by a Cambridge Filter Pad at the same regime (AIR control). Moreover, two negative controls, consisting of one seeded insert with media submerged (INC) and one seeded insert without apical media (ALI) in the incubator, and one positive control with 1 ml apical and 2 ml basal sodium dodecyl sulphate (SDS) at 350 µM were included for each set of exposure. After each exposure, the inserts were transferred from the chamber to a clean well plate, adding 1 mL and 0.5 mL of supplemented UltraCULTURE™ respectively at the basal and apical side for 24 h of recovery period. The recovery period was not performed for the xCELLigence Real-Time Cell analysis.

### Test products and exposure regimens

In this study, we used a standardized experimental tobacco cigarette, 1R6F (University of Kentucky) and four different electronic cigarette liquid samples containing 20 mg/ml of nicotine (hereinafter referred to as “e-liquids”) tested in our laboratories: “Country” (Sample A), “Black Jack” (Sample B), “Artic” (Sample C) and “Desert” (Sample D). All these e-liquids are produced and marketed in Italy by Puff S.r.l. Moreover, a solution containing only e-liquids base (PG/VG; 1:1) and a base solution (PG/VG; 1:1) with nicotine 20 mg/ml (PG/VG NIC) were used as controls. The e-liquids and base solutions (with and without nicotine) were tested using the device Zeep, a puff activated Pod system e-cigarette with a 1.4 ml capacity tank containing a steel 1.2-ohm coil and mounting a 500mAh battery with non-adjustable power and airflow. Mainstream smoke from 1R6F was generated by using the LM1 smoking machine (Körber Technologies GmbH) following the ISO 20778 (ISO 20778: 2018) regimen, which ensures a 55 ml, 2 s duration bell shaped profile, puff every 30 s (55/2/30) with filter hole vents blocked. Whereas, e-cigarette aerosol was produced by using the LM4E vaping machine (Körber Technologies GmbH) under the ISO 20768 (ISO 20768: 2018) regimen (55 mL puff volume, drawn over 3 s, once every 30 s with square shaped profile).

For cell-free ROS evaluation a range of puff numbers was applied in order to evaluate dose-related effects: 9 (1 cigarette), 18 (two cigarettes), 36 (three cigarettes), and 45 puffs (four cigarettes) for 1R6F cigarettes, and 20, 40, 60, and 80 puffs for e-cigarettes. For cytotoxicity (NRU, Annexin V, and RTCA) and mitochondrial potential evaluations, H292 cells were exposed to 5 puffs of 1R6F cigarette smoke, corresponding to the IC50 previously established in similar models [9], or to 10 puffs of e-cigarette aerosol, selected to deliver a comparable amount of nicotine [11]. To perform ALI exposures, smoking and vaping devices were connected to exposure chambers containing Transwell^®^ inserts with cells, which were maintained at 37 °C in a fully visible incubator (SI60 Incubator; Cole-Parmer, Staffordshire, UK) for the entire duration of exposure.

### Reactive oxygen species (ROS) production by aerosol

The e-liquids (A, B, C and D), the 1R6F combustible cigarette, the PG/VG and PG/VG containing nicotine were tested to assess the production of ROS in aerosol by a simple “cell-free” assay. An increasing number of puffs from each of the products mentioned above were bubbled in PBS with Dichlorofluorescin (DCF) and the fluorescence was measured by a fluorimeter. The results were calculated in H_2_O_2_ equivalents by comparing the obtained fluorescence with a standard curve based on increasing concentrations of H_2_O_2_.The experiment was conducted for different numbers of puff (20–80) and laboratory-air bubbled PBS was used as basal control.

### Assessment of cytotoxicity by neutral red uptake (NRU) assay

UltraCULTURE™ medium was removed after 24 h recovery period, and cells were washed twice with PBS. Then, cells were incubated with Neutral Red (NR) dye (0.05 g/L in UltraCULTURE™) for 3 h at 37 °C, 5% CO_2_ in a humidified atmosphere. Subsequently, cells were washed twice with PBS to remove unincorporated dye. 500 µl of destain solution (50% ethanol, 49% distilled water, 1% glacial acetic acid; V:V: V) was added to each insert and incubated for 10 min at 300 rpm on a plate shaker in order to elute incorporated NR from cells. NR extracts were transferred to a 96-well plate in triplicate, in aliquots of 100 µl per well. The optical density of NR extracts was read by a microplate spectrophotometer (Synergy HT, BioTek) at 540 nm using a reference filter of 630 nm. A blank insert (without cells) was used to assess the ability of NR solution to stain the Transwells^®^ membranes. Background measurement from Blank was subtracted from each measurement. NRU levels of treated cells were expressed as a percentage of air-exposed controls.

### Assessment of cytotoxicity by Annexin V apoptosis assay

Evaluation of apoptosis and necrosis was performed using the Muse^®^ Annexin V & dead cell Kit (Luminex Corporation, Austin - USA). After the recovery period of 24 h, NCI-H292 cells were washed, trypsinized (0.25% trypsin) and resuspended in supplemented RPMI-1640 medium. Each exposure condition was analyzed in duplicate following the manufacturers’ instructions. Viable cells [Annexin V-PE (–) and 7AAD (–)], early apoptotic cells [Annexin V-PE (+) and 7AAD (–)], advanced apoptotic cells [Annexin V-PE (+) and 7AAD (+)], and dead cells [Annexin V-PE (–) and 7AAD (+)] were evaluated as percentage gated. The percentage of viable cells was expressed as percentage of AIR control.

### Assessment of cytotoxicity by real-time cell analysis (RTCA)

After the exposure to smoke, aerosol and air, cell proliferation was evaluated using xCELLigence RTCA DPsystem (Agilent, CA, USA). At the end of each exposure, cells were washed twice with PBS, trypsinized (0.25% trypsin), counted and resuspended in supplemented RPMI-1640. Then, cells were seeded in E-16 xCELLigence plate (Agilent, CA, USA) at a density of 15 × 10^3^ cells/ml per well. The plates were subsequently incubated at 37 °C, 5% CO_2_ for 30 min in order to allow cell settling and then placed in the xCELLigence system into a dedicated incubator at the same environmental conditions. Real-time changes in electrical impedance were measured and expressed as “cell index”, defined as (Rn-Rb)/15, where Rb is the background impedance and Rn is the impedance of the well with cells. The background impedance was measured in E-plate 16 with 100µL medium (without cells) after 30 min incubation period at room temperature. Cell proliferation was monitored every 20 min for 71 h.

### Assessment of mitochondrial potential by high content screening (HCS) system

In addition to cytotoxicity, we also evaluated other parameters using HCS: cell morphology and mitochondrial potential. All experiments were performed in triplicate and the results were collected and analyzed by statistical analysis. Mitochondrial potential was assessed by the cationic dye JC-1, which exhibits potential-dependent accumulation in mitochondria. Briefly, after smoke and aerosol exposure, cells were detached and seeded in a 96-wells multiplate (Cell carrier ultra; PerkinElmer) at a density of 10 × 10^3^ cells/well. After 24 h cells were incubated with media containing JC-1 dye (1 µg/ml) for 1 h, which permeates the mitochondria, and with NucBlue Nucleic Acid Stain (Life Technologies Corp.; Eugene, OR, USA; R37605; 2 drops/ml; excitation/emission of 652/669 nm), which is able to enter into the live/dead cells and bind to nucleic acids. After incubation with dyes, cells were washed twice and then read under confocal conditions using the 20x long WD objective by High Content Screening (HCS) analysis system (PerkinElmer Operetta High-Content Imaging System) for 72 h.

### Statistics

All data were assessed for their distribution by using the Shapiro-Wilk normality test. One-way ANOVA followed by Tukey’s post hoc test was performed to analyze differences among the tested products for NRU assay. For RTCA and Annexin V comparisons, p values were calculated by applying two-way ANOVA with differences between groups determined using respectively Dunnett’s and Tukey’s adjustments for multiple comparisons. Moreover, comparisons of JC1 results were analyzed by fitting a repeated measure mixed model followed by Tukey’s test adjustment for multiple comparisons. Data were expressed as mean ± standard error of the mean (SEM), unless otherwise stated. All analyses were considered significant with a p-value of less than 5%. We analyzed and plotted the results using GraphPad Prism version 8 (GraphPad Software, San Diego, California, USA, https://www.graphpad.com/).

## Results

### Nano-microplastics and trace elements

The analysis of NPs and MPs showed slight plastic debris smaller than 10 mm in size in any of the analyzed samples, from 51 particles per gram of e-liquid (p/g) in sample B, to 301 p/g in sample A, to 502 p/g in sample D and, finally, to 1070 p/g in sample C. The analysis of 14 trace elements was performed in all the samples of e-liquid (A, B, C and D) and results are shown in Table 1.

**Table 1:** Concentrations (µg/L) of trace elements in e-liquid (A, B, C, D) compared to reference values established by WHO for drinking-water [12]

Trace element	Ref*	Sample A	Sample B	Sample C	Sample D
Al	100	7.8	7.6	5.6	8.8
As	10	< 1.1	2.5	1.5	2.9
B	500	< 0.1	< 0.1	< 0.1	< 0.1
Cd	3	< 0.5	< 0.5	< 0.5	< 0.5
Cr	50	< 0.5	< 0.5	< 0.5	< 0.5
Cu	2000	< 1.1	< 1.1	< 1.1	< 1.1
Fe	300	110	97	98	76
Hg	6	< 0.5	< 0.5	< 0.5	< 0.5
Mn	100	< 1.5	< 1.5	< 1.5	< 1.5
Ni	70	1.4	< 0.5	1.0	1.2
Pb	10	< 0.5	< 0.5	< 0.5	< 0.5
Sb	5	< 0.5	< 0.5	< 0.5	< 0.5
Se	40	0.8	0.9	0.7	0.98
V	–	< 1.1	< 1.1	< 1.1	< 1.1

Metallic elements considered systemic toxicants in all their chemical forms (As, Hg and Pb) or only some (inorganic As and Cr (VI)), were found below the limit of detection in all the analyzed samples, with the exception of As in samples B, C and D where we found very small amounts, 2.5, 1.5 and 2.9 µg/L respectively, in any case lower than the limit suggested by the WHO for drinking water intended for human consumption [12]. For some elements considered essential, and respect of which there is a very narrow range of concentrations between beneficial and toxic effects, we found concentrations below the LOD or close to it in the case of Ni in samples A, C and D, and Se in all the analyzed samples. Fe had the highest concentration, in the range from 76 to 110 µg/L, in any case lower than the limit suggested by the WHO for drinking water intended for human consumption [12]. Sb had no biological function and was found below LOD in all analyzed samples.

### Reactive oxygen species (ROS) formation

Our results on ROS formation reported in Table 2, showed that PG/VG without nicotine did not generate ROS. 

**Table 2:** ROS values in the cell free assay system. Data are presented as the mean ± standard deviation and referred to equivalents of H_2_O_2_

		20 Puffs	40 Puffs	60 Puffs	80 Puffs
Air Control		–	–	–	–
PG/VG		–	–	–	–
PG/VG + Nicotine 20 mg/ml		–	–	273.1 ± 20.47	519.12 ± 81.86
A		–	–	–	–
B		–	–	–	–
C		–	–	–	–
D		–	–	–	–
	**1 CIG** **(9 Puffs)**	**2 CIG** **(18 Puffs)**	**3 CIG** **(27 Puffs)**	**4 CIG** **(36 Puffs)**	**5 CIG** **(45 Puffs)**
1R6F	508.85 ± 15.65	839.25 ± 40.03	980.85 ± 30.52	1327.53 ± 19.2	1885.8 ± 144.24

Data from e-cigarette and 1R6F combustible cigarettes, which are differently obtained, but referred to the same parameter (ROS). So, we highlighted in bold the data referred to the 1R6F cigarette (1 cig, 2 cigs, etc.) in order to differentiate these data from the previous data on e-cigarettes that were measured only in puff number (20, 40, 60, 80 puff).

Whereas, the use of a PG/VG formulation containing nicotine (20 mg/ml) generated ROS in direct proportion to the number of puffs performed from 60 puffs. The four tested e-liquids (sample A, B, C and D) did not generate ROS under the same experimental conditions. In contrast, 1R6F cigarette smoke generated a large amount of ROS in a dose-dependent manner as early as 9 puffs.

### Effects of e-liquids on cell viability evaluated by NRU assay

**Fig. 1:**
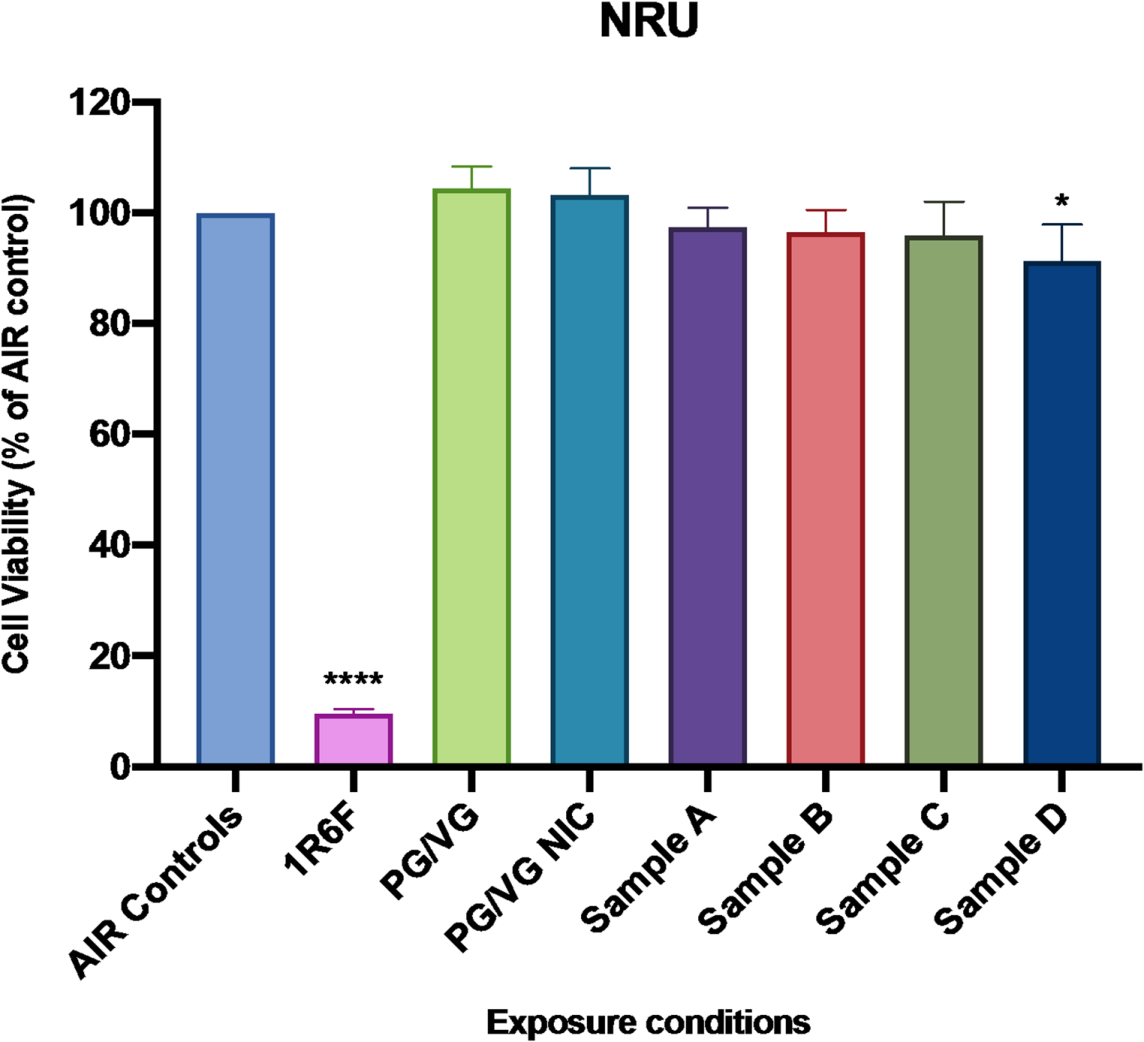
Evaluation of cell viability by NRU assay at 24 h. Cell viability of each tested product is expressed as percentage of its AIR control. The mean ± SEM values were respectively, 9.52 ± 0.37% for 1R6F, 104.4 ± 1.59% for PG/VG, 103.2 ± 1.97% for PG/VG NIC, 97.36 ± 1.48% for Sample A, 96.48 ± 1.63% for Sample B, 95.98 ± 2.46% for Sample C, and 91.25 ± 2.71% for Sample D. Significance code: *p* < 0.0001 (****) vs. AIR control; *p* < 0.05 (*) vs. AIR control

Comparison of NRU cell viability results among all tested products at 24 h from the ALI exposure showed a significant difference with an overall p value < 0.0001 between the reference and the tested e-liquids (Fig. 1).

Substantial reduction of cell viability was observed for cells exposed to 5 puffs of 1R6F smoke (32.02% ± 1.78) compared to AIR control (*p* < 0.0001). A slight significant reduction was also shown for sample D compared to AIR control (*p* < 0.022). No significant differences were observed for PG/VG, PG/VG NIC, sample A, B and C compared to AIR Control.

### Effects of e-liquids on cell viability evaluated by cytofluorimetric Annexin V assay

**Fig. 2:**
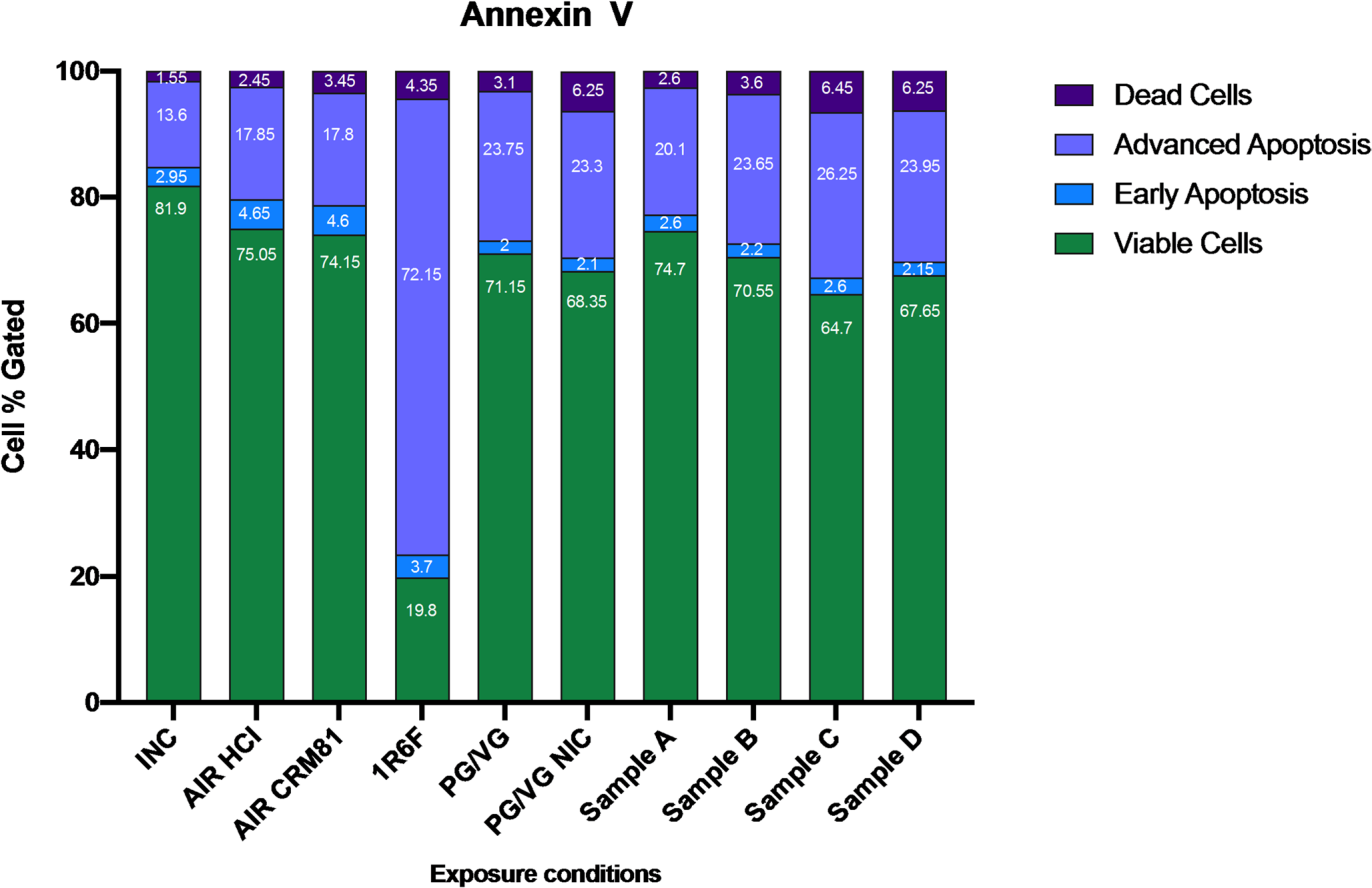
Evaluation of apoptosis and necrosis after smoke/vapor exposures. Each bar in the chart represents the whole (100%) of cell gated for the tested products, and segments in each bar represent the mean percentage of viable cells, early apoptotic cells, advanced apoptotic cells, and dead cells. Data were reported as percentage of cell gated

The cytofluorimetric Annexin V assay allowed the quantification of live cells from cells in early apoptosis, advanced apoptosis, and dead cells, as seen in Fig. 2.

Exposure to 1R6F cigarette smoke showed a strong reduction of viable cells compared to AIR control (*p* = 0.005), PG/VG (*p* = 0.003), PG/VG with nicotine (*p* = 0.016), Sample A (*p* = 0.015), Sample B (*p* = 0.012), Sample C (*p* = 0.015), and Sample D (*p* = 0.014). No differences in viable cells were shown for PG/VG, PG/VG with nicotine, Sample A, Sample B, Sample C, and Sample D compared to AIR control. Whereas, a substantial increase of advanced apoptosis cells was observed after exposure to 1R6F smoke compared to AIR control (*p* < 0.0001), PG/VG (*p* < 0.0001), PG/VG with nicotine (*p* < 0.0001), Sample A (*p* = 0.005), Sample B (*p* < 0.0001), Sample C (*p* < 0.0001), and Sample D (*p* = 0.001). No differences in advanced apoptotic cells were shown for PG/VG, PG/VG with nicotine, Sample A, Sample B, Sample C, and Sample D compared to AIR control. Also, no changes in early apoptotic and dead cells were observed among all the tested exposure conditions.

### Effects of e-liquids on cell viability evaluated by RTCA

**Fig. 3:**
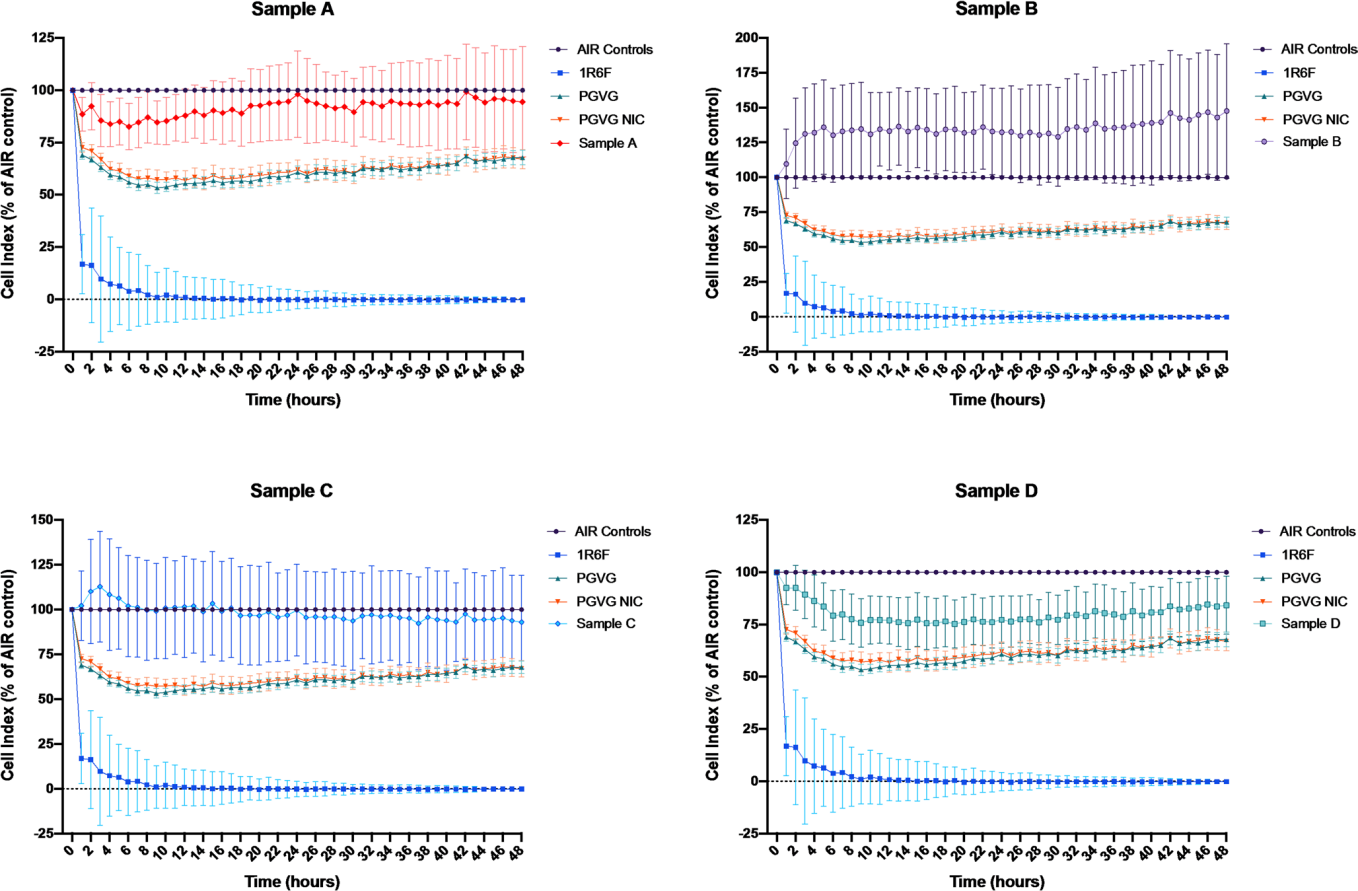
Real-Time Cell Analysis after smoke and vapor exposures. Data are reported as a percentage of the AIR control. The values indicated in the graph refer to mean ± SEM of the electrical resistance (Cell index) which is directly proportional to the number of cells

The RTCA (xCELLigence) results showed that 1R6F cigarette smoked for 5 puffs caused complete cell death as early as 8 h after exposure, exhibiting a significant difference compared to AIR control (*p* < 0.0001). PG/VG and PG/VG NIC cell viability were reduced by approximately 40% at 24 h and 35% at 48 h compared to AIR control (*p* < 0.0001). Whereas, e-liquids seem to affect cell viability less than PG/VG and PG/VG NIC (Fig. 3).

Cells exposed to sample A had a slight reduction in viability in the first 20 h (no more than 10%) with a recovery around 100% viability until 48 h, but a significant difference was observed compared to AIR control (*p* = 0.003). Sample B generates an increase in viability, surpassing even the AIR control from the early hours to 48 h (*p* < 0.0001). Next, Sample C aerosol had a cell viability curve similar to AIR control (*p* = 0.999). Finally, Sample D reduced cell viability by only 25% at 24 h, and 20% at 48 h compared to AIR control (*p* < 0.0001). Though, this reduction is less than 1R6F, PG/VG and PG/VG with nicotine.

### Assessment of mitochondrial potential

**Fig. 4:**
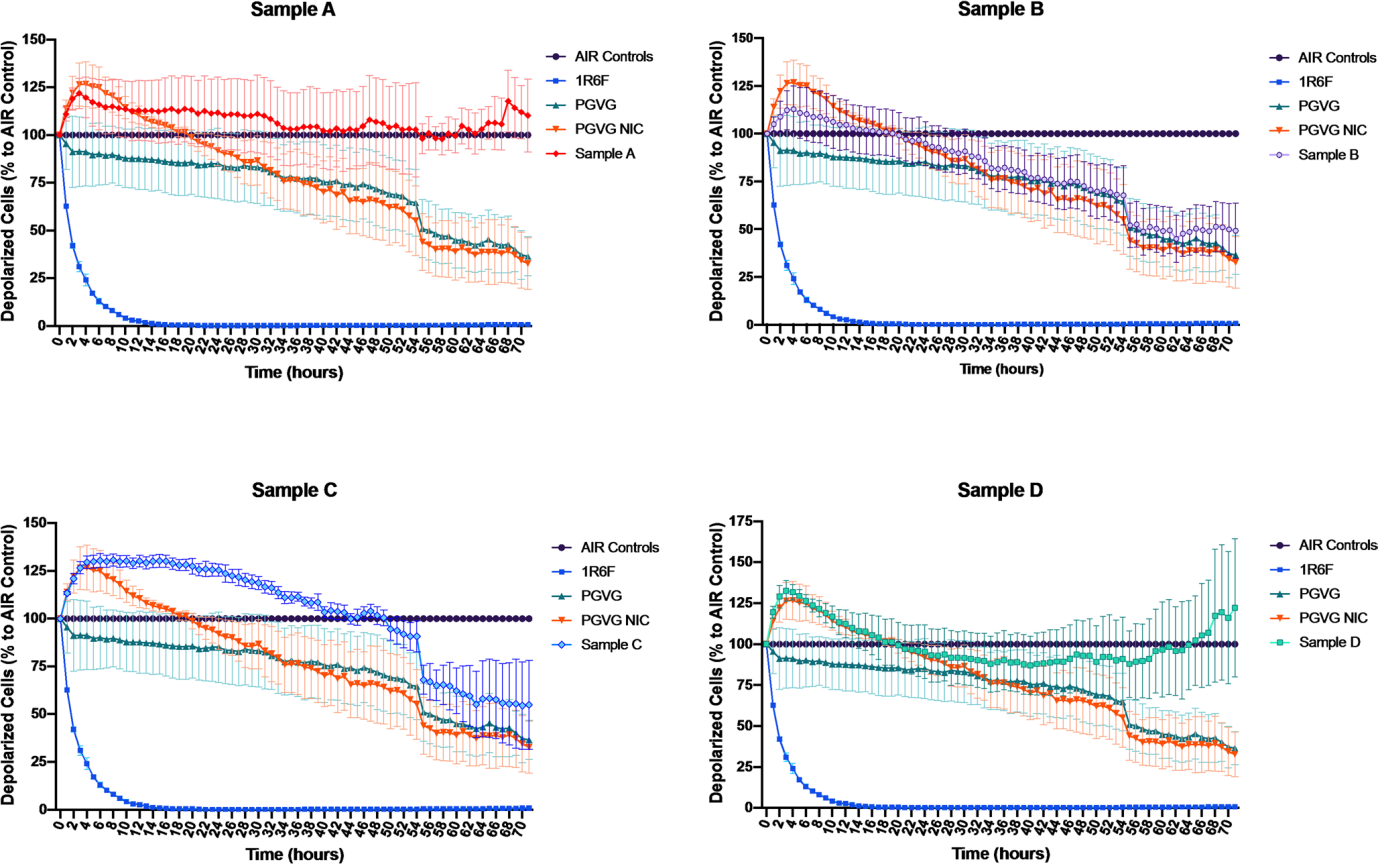
Mitochondrial potential assessment by JC-1 probe. Data are reported as percentage of number of depolarized cells. The values are expressed as mean ± SEM percentage of AIR control

The assessment of mitochondrial function by studying mitochondrial membrane depolarization with fluorescent JC1 probe in HCS showed that all products tested did not cause significant reduction in mitochondrial function, except in cells exposed to 1R6F cigarette smoke (Fig. 4).

1R6F cigarettes generate already at 12 h a strong and long-lasting reduction in mitochondrial function in cells exposed to smoke compared to AIR control (*p* = 0.0002), whereas PG/VG aerosols without nicotine have little effect in the first 24 h, increasing over time to about 50% at 72 h but no significant difference was observed compared to AIR control (*p* = 0.63). Of note, PG/VG NIC-stimulated cells have a peak growth in mitochondrial function at around 6 h, which was depleted around 18–19 h, and then aligned with the reduction measured following stimulation with PG/VG. Likewise, no difference was shown for PG/VG with nicotine compared to AIR control (*p* = 0.811).

Cells exposed to the four e-liquids (Sample A, B, C and B), all containing nicotine (20 mg/ml), show the same initial peak in mitochondrial activity, which reaches variable values and effect duration depending on the flavor mix (Fig. 4). Particularly, sample A generated a relatively low peak of mitochondrial activity, which is maintained for about 24 h by the cells and then returns to a condition similar to AIR control (*p* > 0.999). During the first 13 h, Sample B showed a lower mitochondrial activity peak than PG/VG NIC while the depolarization curve trends became similar during the following hours. Similarly, no difference was shown for Sample B compared to AIR control (*p* = 0.901). Sample C showed an increased mitochondrial depolarization with a longer duration (about 40 h) than PG/VG with nicotine but no difference was observed compared to AIR control (*p* > 0.999). Finally, Sample D produced a slightly higher peak of mitochondrial depolarization than PG/VG NIC, dropping until 37–38 h. A second increase of mitochondrial depolarization after exposure to Sample D was observed around 58 h until 72 h. No difference was observed between Sample D and AIR control (*p* > 0.999).

## Discussion

In this work we evaluated the quality of four e-liquids very popular in Italy, assessing the presence of some contaminants derived from plastics (MPs and NPs) or metals, which could both derive from the processes of production, boxing and transportation to the points of sale. We found a certain quantity of MPs and NPs, from 51 to 1070 p/g, but these values are extremely low considering that these particles are ubiquitous and that the levels found in bottled drinking water obtained with the same method by Zuccarello et al. [13] ranged between 3.16E + 04 p/g and 1.1E + 05 p/g with a main value of 5.42E + 04 p/g (SD 1⁄4 ± 1.95E + 04 p/L), therefore from three to 100 times of the maximum value found in our samples.

Regarding the presence of trace elements, the four liquids analyzed have a good safety profile as products intended for human consumption. Considering that there is not a list of limit values for trace elements in e-liquids yet, we have compared the values measured with those defined as safe by the WHO for drinking water [12] (Table 1). While WHO drinking water limits were used as reference thresholds for detected metal and microplastic levels, we acknowledge that these are based on oral exposure and may not directly reflect inhalation risks. This comparison is only intended as a contextual parameter in the absence of inhalation-specific regulatory values. Among the metallic elements the aluminum (Al) showed values significantly below the limit established for drinking water (from 5.6 µg/L for sample C to 8.8 µg/L for sample D versus 100 µg/L established for drinking water). Aluminum is considered as safe for human health within certain quantities. The FDA has determined that aluminum used as food additives and medicinal is generally safe. Moreover, the United States Environmental Protection Agency (EPA) has recommended a Secondary Maximum Contaminant Level (SMCL) of 0.05–0.2 mg/L for aluminum in drinking water [12]. In tested e-liquids we found concentrations from 5 to 20 times lower than the maximum limits for drinking water. The metallic element that we have detected with the most relevant concentration was Iron (Fe). Concentrations of iron in drinking-water are normally less than 300 µg/L [12], and therefore about three-four times higher than the concentrations found in the e-liquids tested in this study (76–100 µg/L). The other elements assessed, including those linked to mitochondrial dysfunction and DNA fragmentation such as copper [14], were all under the Limit of detection (LOD) or extremely low to represent a possible risk to human health (see Table 1).

Consistently with the results discussed so far, all tested e-liquids showed an excellent safety profile: we evaluated whether these e-liquids when vaporized were able to produce an appreciable amount of ROS, considered as reliable predictors of e-liquid-induced cellular toxicity [15], compared to those produced by the smoke of a classic cigarette. In particular, ROS production in this study was assessed using a cell-free system, which is an established method for evaluating the oxidative potential of complex aerosols [16–18]. This approach allows for sensitive detection of redox activity while minimizing biological variables. However, it is worth noting that, since aerosols-induced cellular response to oxidative stress represents an additional important factor to be assessed, future studies will aim to integrate both acellular and cellular assays for a more complete evaluation. Although the PG/VG e-liquid with nicotine produces a certain amount of ROS during the vaporization by e-cig, possibly due to the pyrolysis effect of nicotine, all the samples (A, B, C and D) did not produce ROS under the conditions tested in the cell-free system assay, suggesting that the aromatic compounds of the products can offset the production of ROS. Moreover, when used with the settings of resistance and power detailed in methods with the “Zeep” e-cigarette, all the products did not show significant and permanent alterations of the cellular metabolism, even in the longest periods of observation (48 h). We exposed bronchial epithelial cells by an air-liquid interface method as this is the most physiologically relevant for bronchial epithelial cell lines, exposing them to all fractions and components of smoke/vapor [19]. The cytotoxic effect at 24 h induced by the aerosol produced with these e-liquid samples is greatly reduced compared to that induced by the smoke of a classic cigarette, both by the classic NRU assay and by the Annexin V apoptosis assay. Consistently, previous studies have shown that e-cigarette aerosol induces significantly less cytotoxicity compared to conventional cigarette smoke [20, 21]. By NRU assay we observed a viability of cells less than 20% at 24 h with ALI exposure to 5 puffs of smoke from 1R6F tobacco cigarette and higher than 90% after 24 h from exposure to e-cig aerosols from all the tested e-liquids. By Annexin V apoptosis assay we had the opportunity to look at the effects of smoking and aerosol more in depth, distinguishing the viable cells in two different populations: a population of “healthy viable cells” and a population of “suffering/dying viable cells” (early apoptosis) (Fig. 2). As a confirmation of concept already demonstrated in a previous multicenter study [22], even with this method the results were clearly in favor of cells exposed to e-cig aerosols compared to those exposed to cigarette smoke, counting a total of 19.8% of viable cells after exposure to smoke and a range from 64.7% (sample C) to 74.7% (sample A) of viable cells after exposure to e-liquids aerosol, therefore with a cytotoxic effect of e-cig aerosol compared to cigarette smoke less than 50% to 60%. Interestingly, it is not clear which fate the suffering cells will follow in early apoptosis, that could either recover or worsen to cell death. This aspect is significantly important because this cell population represents on average 23.5% of the cells exposed to aerosols (both PG/VG and flavored e-liquids) and, even, 72.15% of the cells exposed to smoke at 24 h. This information can be deepened with the RTCA analysis (xCELLigence technology; Agilent, CA, USA), which is able to assess the kinetics of cytotoxicity induced by these products for 72 h after the exposure. The xCELLigence is designed for monitoring cell adhesion and growth. The system exploits microplates with gold electrodes on the bottom of the wells, such that an electric potential is applied across wells. Therefore, the adhering cells to a well reduce the degree of electrons able to flow freely across the established potential (electrical impedance) [23]. Here we have observed a rapid and drastic decline in the growth capacity of cells exposed to cigarette smoke, already in the hours following the treatment. On the other hand, in the exposure of the cells to the aerosol produced by PG/VG and PG/VG + Nicotine, a slight slow and progressive decline in the cell growth capacity is observed in the first hours after exposure to the aerosol and then a slow and progressive recovery up to at 48 h. Finally, flavored e-liquids with nicotine (sample A, B, C and D) do not show significant deviations from the trend of cells exposed only to air, thus highlighting an insignificant effect of e-liquid aerosols on cells up to 48 h after exposure. Mitochondrial potential was also assessed by the cationic dye JC-1, that exhibits potential-dependent accumulation in mitochondria, indicated by a fluorescence emission shift from green (~ 525 nm) to red (~ 590 nm). In particular, JC-1 staining primarily reflects changes in membrane potential, while not providing specific hints on mitochondrial metabolism. Mitochondrial depolarization is indicated by a decrease in the red/green fluorescence intensity ratio. The potential-sensitive color shift is due to concentration dependent formation of red fluorescent aggregates. The ratio of green to red fluorescence is dependent only on the membrane potential. Comparative measurements of membrane potential allow to determine the percentage of mitochondria able to respond to an applied stimulus and to highlight the mitochondrial depolarization occurring in the early stages of apoptosis. This method was also useful to better decrypt the effect of smoke and aerosol on cells throughout the 71 h following the exposure, and therefore to evaluate the ability of these products to disrupt cellular metabolism. Although recent findings suggest that e-cigarette vapor can induce mitochondrial stress and alter cellular respiration pathways [24, 25], our in vitro results, performed with standardized methods for e-cig vapor exposure on cells, showed an opposite trend. The sample A, C and D showed slight or no relevant effect on mitochondria, otherwise sample B showed a slow and gradual depolarization of cells over the time, while always remaining above the depolarization observed with cigarette smoke. On the contrary, cigarette smoke after 14 h is able to completely depolarize the mitochondria. In this context, and in order to provide a more comprehensive assessment, future studies should integrate additional functional assays, such as ATP quantification or respiratory complex activity analysis, to further characterize mitochondrial impairment. The results of the study indicate lower metabolic perturbation at mitochondrial level, no production of reactive oxygen species, and a substantially reduced cytotoxic effect of e-liquid aerosol compared to cigarette smoke. Under normal condition of use and with the power settings recommended by manufacturers, the vaping products under investigation proved to be significantly less harmful to human cell systems compared to conventional cigarettes.

This study has some limitations that should be acknowledged. First, cytotoxicity was assessed at a single exposure level (5 puffs of 1R6F smoke and 10 puffs of e-cigarette aerosol, corresponding to comparable nicotine release), which enabled a biologically meaningful comparison but did not allow assessment of dose–response relationships or precise toxicity thresholds. While ROS production was assessed in an acellular system that allowed testing a growing range of puffs to analyze dose-dependent release, cytotoxicity was investigated in cell cultures at a single exposure level. This difference in experimental design reflects the specificity of the two assays, but also constitutes a limitation, as it does not allow dose-response relationships for cytotoxicity to be established. Second, WHO drinking water guidelines were used as reference values for trace elements and microplastics due to the absence of inhalation-specific safety thresholds; these values should therefore be interpreted with caution, as they do not directly reflect inhalation exposure risks. Finally, although both cell-free and cellular assays were employed, additional endpoints, including long-term cellular responses, will be important to further elucidate the toxicological profile of e-cigarette aerosols.

## Conclusions

This study is a further confirmation that products alternative to tobacco cigarette, even if tested on different cell lines and investigated with different methods, have effects on cell physiology, but dramatically decreased if compared to traditional cigarettes. Our results support the reduced harmful potential of e-cigs relative to tobacco cigarettes in an in vitro model of human bronchial epithelial cells, and support the use of ENDS as a viable option in harm reduction strategies for smokers. However, these findings should be interpreted with caution when moving to clinical practice. They represent acute, short-term effects in vitro, and repeated or chronic exposures may exacerbate these responses or reveal additional endpoints, including inflammatory outcomes. Future studies will therefore focus on longer-term exposure models to better meet real-life conditions.

## Data Availability

The datasets generated during and/or analyzed during the current study are available from the corresponding author on reasonable request.
